# Supplementary material for: Increasing childhood illnesses (diarrhea and fever) and decreasing care-seeking practices in Nepal: Insights from three most recent Demographic and Health Surveys (2011, 2016 and 2022)
Source: PLOS Glob Public Health. 2025 Dec 11;5(12):e0005651. doi: 10.1371/journal.pgph.0005651 (PMC12698019; doi:10.1371/journal.pgph.0005651)
Supplement: S1 Table — (DOCX) [file pgph.0005651.s001.docx]

S1 Table: Prevalence of diarrhea and fever among children under five two weeks prior to the survey, NDHS 2022.

| **Characteristics** | **Diarrhea (%)** | **N** | **P** | **Fever (%)** | **N=5,040** | **P** |
| --- | --- | --- | --- | --- | --- | --- |
| **National average** | **10.4** | **5,040** |  | **23** |  |  |
| **Sex of the child** |  |  | 0.617 |  |  | 0.333 |
| Male | 10.6 | 2,639 |  | 23.6 | 2,639 |  |
| Female | 10.1 | 2,401 |  | 22.3 | 2,401 |  |
| **Child age in months** |  |  | <0.001 |  |  | 0.003 |
| <6 | 12.7 | 533 |  | 18 | 533 |  |
| 6–12 | 17.5 | 434 |  | 27.3 | 434 |  |
| 12–23 | 13.2 | 959 |  | 24.3 | 959 |  |
| 24–35 | 9.7 | 1,066 |  | 24.3 | 1,066 |  |
| 36–47 | 6.7 | 1,048 |  | 24.7 | 1,048 |  |
| 48–59 | 8.1 | 1,000 |  | 19.3 | 1,000 |  |
| **Maternal age in years** |  |  | 0.259 |  |  | 0.882 |
| <20 | 12.1 | 994 |  | 22.5 | 994 |  |
| 20–29 | 10 | 3,286 |  | 23.2 | 3,286 |  |
| 30 and above | 9.9 | 761 |  | 22.5 | 761 |  |
| **Religion** |  |  | 0.935 |  |  | 0.433 |
| Hindu | 10.4 | 4,218 |  | 23.2 | 4,218 |  |
| Other | 10.3 | 822 |  | 21.7 | 822 |  |
| **Ethnicity** |  |  | 0.177 |  |  | 0.054 |
| Brahmin | 7 | 374 |  | 26.9 | 374 |  |
| Chhetri | 8.4 | 873 |  | 24.6 | 873 |  |
| Madheshi | 12.1 | 1,005 |  | 20.2 | 1,005 |  |
| Dalit | 12 | 935 |  | 23.8 | 935 |  |
| Janajati | 10.6 | 1,417 |  | 23.3 | 1,417 |  |
| Newar | 11.3 | 119 |  | 29.1 | 119 |  |
| Muslim | 8.4 | 317 |  | 16.5 | 317 |  |
| **Education** |  |  | 0.435 |  |  | 0.063 |
| No education | 11.3 | 1,133 |  | 19.9 | 1,133 |  |
| Basic | 10.7 | 1,737 |  | 22.6 | 1,737 |  |
| Secondary | 9.9 | 1,955 |  | 25.1 | 1,955 |  |
| Higher | 7.4 | 215 |  | 23.3 | 215 |  |
| **Wealth quintile** |  |  | 0.013 |  |  | 0.301 |
| Poorest | 8.9 | 1,213 |  | 21.8 | 1,213 |  |
| Poorer | 11.4 | 1,037 |  | 25.2 | 1,037 |  |
| Middle | 12.7 | 1,048 |  | 23.8 | 1,048 |  |
| Richer | 11.1 | 969 |  | 23.6 | 969 |  |
| Richest | 7.6 | 774 |  | 20.1 | 774 |  |
| **Marginalization status** |  |  | 0.509 |  |  | 0.143 |
| Triple | 10.5 | 615 |  | 19.2 | 615 |  |
| Double | 11.5 | 1,510 |  | 23 | 1,510 |  |
| Single | 9.9 | 2,118 |  | 23.1 | 2,118 |  |
| No | 9.5 | 797 |  | 25.5 | 797 |  |
| **Province** |  |  | 0.403 |  |  | 0.033 |
| Koshi | 11 | 859 |  | 25.7 | 859 |  |
| Madhesh | 10 | 1,352 |  | 19.7 | 1,352 |  |
| Bagmati | 13 | 814 |  | 21.4 | 814 |  |
| Gandaki | 7.8 | 331 |  | 25.8 | 331 |  |
| Lumbini | 9.8 | 862 |  | 23 | 862 |  |
| Karnali | 10.4 | 371 |  | 28 | 371 |  |
| Sudurpashchim | 8.8 | 451 |  | 24.1 | 451 |  |
| **Residence** |  |  | 0.103 |  |  | 0.517 |
| Urban | 11.1 | 3,276 |  | 23.3 | 3,276 |  |
| Rural | 9.1 | 1,764 |  | 22.3 | 1,764 |  |
| **Ecological region** |  |  | 0.059 |  |  | 0.005 |
| Mountain | 6.9 | 317 |  | 20.3 | 317 |  |
| Hill | 9.5 | 1,744 |  | 26.1 | 1,744 |  |
| Terai | 11.3 | 2,979 |  | 21.5 | 2,979 |  |
| **Native language** |  |  | 0.933 |  |  | <0.001 |
| Nepali | 10.1 | 2,383 |  | 26.1 | 2,383 |  |
| Maithili | 11 | 1,006 |  | 22.1 | 1,006 |  |
| Bhojpuri | 9.8 | 466 |  | 17.6 | 466 |  |
| Other | 10.7 | 1,185 |  | 19.6 | 1,185 |  |
| **Birth order** |  |  | 0.648 |  |  | 0.085 |
| First | 10.6 | 2,050 |  | 22 | 2,050 |  |
| Second | 10.7 | 1,715 |  | 25 | 1,715 |  |
| Third or higher | 9.6 | 1,275 |  | 21.8 | 1,275 |  |
