# Supplementary material for: Increasing childhood illnesses (diarrhea and fever) and decreasing care-seeking practices in Nepal: Insights from three most recent Demographic and Health Surveys (2011, 2016 and 2022)
Source: PLOS Glob Public Health. 2025 Dec 11;5(12):e0005651. doi: 10.1371/journal.pgph.0005651 (PMC12698019; doi:10.1371/journal.pgph.0005651)
Supplement: S2 Table — (DOCX) [file pgph.0005651.s002.docx]

S2 Table: Distribution of children under five years old who sought care for the treatment of diarrhea, and fever, NDHS 2022.

| **Characteristics** | **Diarrhea (%)** | **N** | **P** | **Fever (%)** | **N=1,159** | **P** |
| --- | --- | --- | --- | --- | --- | --- |
| **National** | **57.2** | **524** |  | **78.1** |  |  |
| **Sex of the child** |  |  | 0.622 |  |  | 0.902 |
| Male | 58.5 | 281 |  | 78.2 | 624 |  |
| Female | 55.6 | 243 |  | 77.9 | 535 |  |
| **Child age in months** |  |  | 0.767 |  |  | 0.892 |
| <6 | 55.1 | 68 |  | 78.9 | 96 |  |
| 6–12 | 53.2 | 76 |  | 80.5 | 118 |  |
| 12–23 | 55.9 | 126 |  | 79.1 | 233 |  |
| 24–35 | 63.5 | 103 |  | 79.2 | 259 |  |
| 36–47 | 61.6 | 71 |  | 77 | 259 |  |
| 48–59 | 52.7 | 81 |  | 74.9 | 193 |  |
| **Maternal age in years** |  |  | 0.97 |  |  | 0.493 |
| <20 | 56.1 | 121 |  | 79 | 224 |  |
| 20–29 | 57.5 | 329 |  | 78.7 | 764 |  |
| 30 and above | 57.4 | 75 |  | 74.1 | 171 |  |
| **Religion** |  |  | 0.291 |  |  | 0.919 |
| Hindu | 58.3 | 440 |  | 78.1 | 981 |  |
| Other | 51.2 | 85 |  | 77.7 | 178 |  |
| **Ethnicity** |  |  | 0.094 |  |  | <0.001 |
| Brahmin | 72.4 | 26 |  | 88.2 | 101 |  |
| Chhetri | 61 | 74 |  | 74.2 | 215 |  |
| Madheshi | 57.8 | 122 |  | 84.6 | 203 |  |
| Dalit | 58 | 112 |  | 78 | 223 |  |
| Janajati | 53.9 | 150 |  | 74.4 | 331 |  |
| Newar | 18 | 13 |  | 52.3 | 35 |  |
| Muslim | 64.1 | 27 |  | 89.9 | 52 |  |
| **Maternal education** |  |  | 0.056 |  |  | 0.456 |
| No education | 59.5 | 128 |  | 82.2 | 226 |  |
| Basic | 55.4 | 186 |  | 76.9 | 393 |  |
| Secondary | 54.7 | 194 |  | 77.6 | 490 |  |
| Higher | 90.3 | 16 |  | 72.8 | 50 |  |
| **Wealth quintile** |  |  | 0.245 |  |  | <0.001 |
| Poorest | 49.5 | 107 |  | 68.6 | 264 |  |
| Poorer | 64.9 | 118 |  | 73.9 | 261 |  |
| Middle | 53.5 | 133 |  | 82.4 | 249 |  |
| Richer | 56 | 107 |  | 88.5 | 228 |  |
| Richest | 66.2 | 59 |  | 79.1 | 156 |  |
| **Marginalization status** |  |  | 0.571 |  |  | 0.182 |
| Triple | 64.9 | 64 |  | 79.1 | 118 |  |
| Double | 53.9 | 174 |  | 74.6 | 348 |  |
| Single | 56.8 | 210 |  | 78 | 489 |  |
| No | 59.3 | 76 |  | 83.6 | 203 |  |
| **Province** |  |  | 0.525 |  |  | 0.004 |
| Koshi | 48.7 | 95 |  | 76.2 | 221 |  |
| Madhesh | 56.8 | 135 |  | 88.7 | 267 |  |
| Bagmati | 59.3 | 106 |  | 75.8 | 174 |  |
| Gandaki | 48.7 | 26 |  | 72.1 | 86 |  |
| Lumbini | 65 | 84 |  | 78.5 | 198 |  |
| Karnali | 56.4 | 38 |  | 70.7 | 104 |  |
| Sudurpashchim | 62.6 | 40 |  | 70.5 | 109 |  |
| **Residence** |  |  | 0.686 |  |  | 0.061 |
| Urban | 56.5 | 364 |  | 79.9 | 765 |  |
| Rural | 58.7 | 161 |  | 74.6 | 394 |  |
| **Ecological region** |  |  | 0.405 |  |  | <0.001 |
| Mountain | 50.8 | 22 |  | 71.3 | 64 |  |
| Hill | 53.6 | 166 |  | 69.4 | 455 |  |
| Terai | 59.4 | 336 |  | 84.9 | 639 |  |
| **Native language** |  |  | 0.905 |  |  | 0.013 |
| Nepali | 55.8 | 241 |  | 75.2 | 621 |  |
| Maithili | 56.1 | 111 |  | 84.2 | 222 |  |
| Bhojpuri | 61.7 | 46 |  | 88.9 | 82 |  |
| Other | 59 | 127 |  | 76.1 | 233 |  |
| **Birth order** |  |  | 0.011 |  |  | 0.838 |
| First | 62.3 | 218 |  | 77.2 | 451 |  |
| Second | 47.6 | 183 |  | 78.9 | 429 |  |
| Third or higher | 62.3 | 123 |  | 78.3 | 278 |  |
