# Supplementary material for: Increasing childhood illnesses (diarrhea and fever) and decreasing care-seeking practices in Nepal: Insights from three most recent Demographic and Health Surveys (2011, 2016 and 2022)
Source: PLOS Glob Public Health. 2025 Dec 11;5(12):e0005651. doi: 10.1371/journal.pgph.0005651 (PMC12698019; doi:10.1371/journal.pgph.0005651)
Supplement: S3 Table — (DOCX) [file pgph.0005651.s003.docx]

S3 Table: Distribution of children under five years old who had diarrhea two weeks prior to the survey and sought the treatment of diarrhea, and fever private HFs, NDHS 2022.

| **Characteristics** | **Diarrhea (%)** | **N=300** | **P** | **Fever (%)** | **N=904** | **P** |
| --- | --- | --- | --- | --- | --- | --- |
| **National** | **72.7** | **300** |  | 80.4 | 905 |  |
| **Sex of the child** |  |  | 0.95 |  |  | 0.139 |
| Male | 72.5 | 165 |  | 82.2 | 488 |  |
| Female | 72.9 | 135 |  | 78.3 | 417 |  |
| **Child age in months** |  |  | 0.431 |  |  | 0.285 |
| <6 | 69.4 | 37 |  | 82.3 | 76 |  |
| 6–12 | 81.6 | 40 |  | 84.7 | 95 |  |
| 12–23 | 79.6 | 71 |  | 84.7 | 185 |  |
| 24–35 | 65.4 | 66 |  | 76 | 205 |  |
| 36–47 | 71.8 | 43 |  | 80.6 | 199 |  |
| 48–59 | 67.5 | 43 |  | 77.2 | 145 |  |
| **Maternal age in years** |  |  | 0.628 |  |  | 0.959 |
| <20 | 69.3 | 68 |  | 80.7 | 177 |  |
| 20–29 | 74.9 | 189 |  | 80.5 | 601 |  |
| 30 and above | 68 | 43 |  | 79.4 | 127 |  |
| **Religion** |  |  | 0.433 |  |  | 0.638 |
| Hindu | 73.6 | 256 |  | 80.1 | 766 |  |
| Other | 67.3 | 43 |  | 82.3 | 138 |  |
| **Ethnicity** |  |  | 0.005 |  |  | <0.001 |
| Brahmin | 84.6 | 19 |  | 85.1 | 89 |  |
| Chhetri | 53.3 | 45 |  | 65.2 | 159 |  |
| Madheshi | 87.9 | 70 |  | 92 | 172 |  |
| Dalit | 61 | 65 |  | 75 | 174 |  |
| Janajati | 74.7 | 81 |  | 79.7 | 246 |  |
| Newar | 75.7 | 2 |  | 96.7 | 18 |  |
| Muslim | 82 | 17 |  | 98 | 47 |  |
| **Maternal education** |  |  | 0.139 |  |  | 0.368 |
| No education | 77.8 | 76 |  | 76.2 | 186 |  |
| Basic | 63.3 | 103 |  | 82 | 302 |  |
| Secondary | 77.2 | 106 |  | 82 | 380 |  |
| Higher | 79.3 | 14 |  | 72.1 | 36 |  |
| **Wealth quintile** |  |  | <0.001 |  |  | <0.001 |
| Poorest | 42.8 | 53 |  | 52.5 | 181 |  |
| Poorer | 72.5 | 77 |  | 76.8 | 193 |  |
| Middle | 75.3 | 71 |  | 87.6 | 205 |  |
| Richer | 87.2 | 60 |  | 93.1 | 202 |  |
| Richest | 86.6 | 39 |  | 94.4 | 123 |  |
| **Marginalization status** |  |  | 0.638 |  |  | <0.001 |
| Triple | 74.9 | 42 |  | 74.8 | 94 |  |
| Double | 67.8 | 94 |  | 71 | 259 |  |
| Single | 73 | 119 |  | 83.9 | 382 |  |
| No | 79.8 | 45 |  | 90 | 170 |  |
| **Province** |  |  | 0.004 |  |  | <0.001 |
| Koshi | 64.5 | 46 |  | 78.3 | 168 |  |
| Madhesh | 84.3 | 77 |  | 92 | 237 |  |
| Bagmati | 74 | 63 |  | 77.2 | 132 |  |
| Gandaki | 55.5 | 13 |  | 80.3 | 62 |  |
| Lumbini | 86.7 | 55 |  | 89.8 | 156 |  |
| Karnali | 34.2 | 22 |  | 48.7 | 73 |  |
| Sudurpashchim | 59.7 | 25 |  | 66.2 | 77 |  |
| **Residence** |  |  | 0.225 |  |  | <0.001 |
| Urban | 75.2 | 206 |  | 85.4 | 611 |  |
| Rural | 67.2 | 94 |  | 70 | 294 |  |
| **Ecological region** |  |  | <0.001 |  |  | 0 |
| Mountain | 31.6 | 11 |  | 35.5 | 46 |  |
| Hill | 49.9 | 89 |  | 66.5 | 316 |  |
| Terai | 85.1 | 200 |  | 92.3 | 543 |  |
| **Native language** |  |  | 0.045 |  |  | <0.001 |
| Nepali | 61.3 | 135 |  | 74.1 | 467 |  |
| Maithili | 87.1 | 62 |  | 93.9 | 187 |  |
| Bhojpuri | 78.2 | 28 |  | 86.7 | 73 |  |
| Other | 79.1 | 75 |  | 80 | 177 |  |
| **Birth order** |  |  | 0.801 |  |  | <0.001 |
| First | 72.2 | 136 |  | 79.2 | 348 |  |
| Second | 70.8 | 87 |  | 84.9 | 339 |  |
| Third or higher | 75.7 | 77 |  | 75.3 | 218 |  |
