# Supplementary material for: Increasing childhood illnesses (diarrhea and fever) and decreasing care-seeking practices in Nepal: Insights from three most recent Demographic and Health Surveys (2011, 2016 and 2022)
Source: PLOS Glob Public Health. 2025 Dec 11;5(12):e0005651. doi: 10.1371/journal.pgph.0005651 (PMC12698019; doi:10.1371/journal.pgph.0005651)
Supplement: S4 Table — (DOCX) [file pgph.0005651.s004.docx]

S4 Table: Bivariable logistic regression analysis of children under 5 who had diarrhea and fever 2 weeks prior to the survey, NDHS 2022

|  |  | **Diarrhoea** |  | **Fever** |  |
| --- | --- | --- | --- | --- | --- |
| **Variable** | **Categories** | **Crude odds ratio** | **95% CI** | **COR** | **95% CI** |
| **Child age in months** | <6 |  |  | **1** |  |
|  | 6–12 | 1.46 | 0.97–2.20 | 1.71** | 1.23–2.37 |
|  | 12–23 | 1.04 | 0.72–1.51 | 1.47** | 1.11–1.93 |
|  | 24–35 | 0.74 | 0.49–1.11 | 1.47* | 1.09–1.97 |
|  | 36–47 | 0.50** | 0.33–0.76 | 1.50** | 1.12–1.99 |
|  | 48–59 | 0.60* | 0.41–0.89 | 1.09 | 0.81–1.48 |
| **Sex of the child** | Male |  |  | 1 |  |
|  | Female | 0.95 | 0.76–1.17 | 0.93 | 0.80–1.08 |
| **Maternal age** | <20 |  |  | 1 |  |
|  | 20–29 | 0.80 | 0.61–1.06 | 1.04 | 0.87–1.25 |
|  | ≥30 | 0.79 | 0.54–1.17 | 1.00 | 0.77–1.31 |
| **Religion** | Hindu |  |  | 1 |  |
|  | Other | 0.99 | 0.73–1.33 | 0.91 | 0.73–1.15 |
| **Ethnicity** | Brahmin |  |  | 1 |  |
|  | Chhetri | 1.22 | 0.78–1.92 | 0.89 | 0.65–1.21 |
|  | Madheshi | 1.83* | 1.04–3.21 | 0.69* | 0.49–0.97 |
|  | Dalit | 1.81* | 1.10–2.97 | 0.85 | 0.61–1.18 |
|  | Janajati | 1.57 | 0.98–2.52 | 0.83 | 0.60–1.13 |
|  | Newar | 1.69 | 0.73–3.94 | 1.12 | 0.61–2.05 |
|  | Muslim | 1.22 | 0.61–2.45 | 0.54* | 0.32–0.89 |
| **Maternal education** | No education |  |  | 1 |  |
|  | Basic | 0.94 | 0.71–1.25 | 1.17 | 0.95–1.45 |
|  | Secondary | 0.86 | 0.64–1.16 | 1.34** | 1.08–1.67 |
|  | Higher | 0.63 | 0.33–1.21 | 1.22 | 0.77–1.95 |
| **Wealth quintile** | Lowest |  |  | 1 |  |
|  | Second | 1.32 | 0.97–1.79 | 1.20 | 0.97–1.50 |
|  | Middle | 1.49* | 1.09–2.03 | 1.12 | 0.89–1.40 |
|  | Fourth | 1.28 | 0.95–1.73 | 1.10 | 0.87–1.40 |
|  | Highest | 0.85 | 0.57–1.26 | 0.90 | 0.67–1.21 |
| **Disadvantages** | Triple |  |  | 1 |  |
|  | Double | 1.11 | 0.77–1.61 | 1.26 | 0.94–1.69 |
|  | Single | 0.94 | 0.65–1.37 | 1.26 | 0.94–1.69 |
|  | No | 0.90 | 0.57–1.40 | 1.44* | 1.04–1.99 |
| **Province** | Koshi |  |  | 1 |  |
|  | Madhesh | 0.90 | 0.56–1.43 | 0.71* | 0.53–0.94 |
|  | Bagmati | 1.21 | 0.79–1.85 | 0.79 | 0.58–1.07 |
|  | Gandaki | 0.68 | 0.41–1.13 | 1.01 | 0.71–1.43 |
|  | Lumbini | 0.87 | 0.59–1.30 | 0.86 | 0.63–1.18 |
|  | Karnali | 0.93 | 0.66–1.33 | 1.12 | 0.86–1.46 |
|  | Sudurpaschim | 0.78 | 0.51–1.19 | 0.92 | 0.68–1.23 |
| **Place of residence** | Urban |  |  | 1 |  |
|  | Rural | 0.80 | 0.62–1.05 | 0.94 | 0.79–1.12 |
| **Ecoregion** | Mountain |  |  | 1 |  |
|  | Hill | 1.43 | 0.98–2.08 | 1.39* | 1.01–1.90 |
|  | Terai | 1.73** | 1.19–2.51 | 1.07 | 0.78–1.47 |
| **Native language** | Nepali |  |  | 1 |  |
|  | Maithili | 1.10 | 0.75–1.61 | 0.81 | 0.64–1.01 |
|  | Bhojpuri | 0.97 | 0.46–2.02 | 0.61*** | 0.45–0.81 |
|  | Other | 1.06 | 0.82–1.38 | 0.69*** | 0.56–0.85 |
| **Birth order** | First |  |  | 1 |  |
|  | Second | 1.01 | 0.81–1.25 | 1.18* | 1.01–1.40 |
|  | Third and higher | 0.90 | 0.68–1.18 | 0.99 | 0.81–1.20 |

* *p*<.05, ** *p*<.01, *** *p*<.001CI = confidence interval
