# Supplementary material for: Increasing childhood illnesses (diarrhea and fever) and decreasing care-seeking practices in Nepal: Insights from three most recent Demographic and Health Surveys (2011, 2016 and 2022)
Source: PLOS Glob Public Health. 2025 Dec 11;5(12):e0005651. doi: 10.1371/journal.pgph.0005651 (PMC12698019; doi:10.1371/journal.pgph.0005651)
Supplement: S5 Table — (DOCX) [file pgph.0005651.s005.docx]

S5 Table: Bivariable logistic regression analysis of children under 5 who had diarrhea and fever and sought care in the 2 weeks prior to the survey, NDHS 2022

|  |  | **Diarrhea** |  | **Fever** |  |
| --- | --- | --- | --- | --- | --- |
| **Variable** | **Categories** | **COR** | **95% CI** | **COR** | **95% CI** |
| **Child age in months** | <6 | **1** |  | **1** |  |
|  | 6–12 | 1.08 | 0.48–2.41 | 1.11 | 0.53–2.29 |
|  | 12–23 | 0.97 | 0.48–1.94 | 1.01 | 0.52–1.97 |
|  | 24–35 | 0.70 | 0.31–1.59 | 1.02 | 0.53–1.95 |
|  | 36–47 | 0.77 | 0.33–1.76 | 0.90 | 0.47–1.71 |
|  | 48–59 | 1.10 | 0.46–2.65 | 0.80 | 0.41–1.55 |
| **Sex of the child** | Male | 1 |  | 1 |  |
|  | Female | 1.13 | 0.70–1.81 | 0.98 | 0.75–1.29 |
| **Maternal age** | < 20 | 1 |  | 1 |  |
|  | 20–29 | 0.95 | 0.59–1.51 | 0.98 | 0.66–1.47 |
|  | ≥30 | 0.95 | 0.48–1.91 | 0.76 | 0.44–1.31 |
| **Religion** | Hindu | 1 |  | 1 |  |
|  | Other | 1.33 | 0.78–2.27 | 0.98 | 0.62–1.54 |
| **Ethnicity** | Brahmin | 1 |  | 1 |  |
|  | Chhetri | 1.67 | 0.57–4.91 | 0.39** | 0.19–0.79 |
|  | Madheshi | 1.91 | 0.73–5.02 | 0.74 | 0.32–1.70 |
|  | Dalit | 1.90 | 0.70–5.16 | 0.48* | 0.23–0.99 |
|  | Janajati | 2.24 | 0.93–5.41 | 0.39** | 0.20–0.75 |
|  | Newar | 11.9** | 2.13–66.36 | 0.15*** | 0.06–0.36 |
|  | Muslim | 1.46 | 0.46–4.64 | 1.19 | 0.40–3.52 |
| **Maternal education** | No education | 1 |  | 1 |  |
|  | Basic | 1.18 | 0.72–1.95 | 0.72 | 0.47–1.10 |
|  | Secondary | 1.22 | 0.72–2.06 | 0.75 | 0.49–1.14 |
|  | Higher | 0.16** | 0.04–0.62 | 0.58 | 0.23–1.47 |
| **Wealth quintile** | Lowest | 1 |  | 1 |  |
|  | Second | 0.53* | 0.29–0.96 | 1.30 | 0.84–2.00 |
|  | Middle | 0.85 | 0.47–1.55 | 2.14** | 1.32–3.47 |
|  | Fourth | 0.77 | 0.43–1.38 | 3.52*** | 2.09–5.91 |
|  | Highest | 0.50 | 0.22–1.13 | 1.73 | 0.96–3.13 |
| **Disadvantages** | Triple | 1 |  | 1 |  |
|  | Double | 1.58 | 0.79–3.13 | 0.78 | 0.44–1.36 |
|  | Single | 1.41 | 0.73–2.70 | 0.94 | 0.54–1.64 |
|  | No | 1.27 | 0.60–2.69 | 1.35 | 0.69–2.65 |
| **Province** | Koshi | 1 |  | 1 |  |
|  | Madhesh | 0.72 | 0.37–1.41 | 2.46** | 1.38–4.38 |
|  | Bagmati | 0.65 | 0.33–1.29 | 0.98 | 0.56–1.73 |
|  | Gandaki | 1.00 | 0.36–2.78 | 0.81 | 0.41–1.58 |
|  | Lumbini | 0.51 | 0.25–1.06 | 1.15 | 0.64–2.06 |
|  | Karnali | 0.73 | 0.35–1.53 | 0.75 | 0.47–1.21 |
|  | Sudurpaschim | 0.57 | 0.29–1.11 | 0.75 | 0.44–1.28 |
| **Place of residence** | Urban | 1 |  | 1 |  |
|  | Rural | 0.92 | 0.60–1.4 | 0.74 | 0.54–1.01 |
| **Ecoregion** | Mountain | 1 |  | 1 |  |
|  | Hill | 0.90 | 0.38–2.08 | 0.91 | 0.57–1.45 |
|  | Terai | 0.71 | 0.31–1.63 | 2.26*** | 1.40–3.64 |
| **Native language** | Nepali | 1 |  | 1 |  |
|  | Maithili | 0.99 | 0.55–1.76 | 1.76* | 1.09–2.84 |
|  | Bhojpuri | 0.78 | 0.38–1.61 | 2.63* | 1.22–5.69 |
|  | Other | 0.88 | 0.51–1.50 | 1.05 | 0.69–1.58 |
| **Birth order** | First | 1 |  | 1 |  |
|  | Second | 1.82** | 1.22–2.71 | 1.10 | 0.79–1.55 |
|  | Third and higher | 1.00 | 0.59–1.68 | 1.06 | 0.73–1.54 |
|  | | | |  |  |

* *p*<.05, ** *p*<.01 CI = confidence interval* *p*<.05, ** *p*<.01, *** *p*<.001CI = confidence interval
