# Supplementary material for: Increasing childhood illnesses (diarrhea and fever) and decreasing care-seeking practices in Nepal: Insights from three most recent Demographic and Health Surveys (2011, 2016 and 2022)
Source: PLOS Glob Public Health. 2025 Dec 11;5(12):e0005651. doi: 10.1371/journal.pgph.0005651 (PMC12698019; doi:10.1371/journal.pgph.0005651)
Supplement: S6 Table — (DOCX) [file pgph.0005651.s006.docx]

S6 Table: Bivariable logistic regression analysis of children under 5 who had diarrhea, and fever and sought care in private HFs in the 2 weeks prior to the survey, NDHS 2022

|  |  | **Diarrhoea** |  | **Fever** |  |
| --- | --- | --- | --- | --- | --- |
| **Variable** | **Categories** | **Crude OR** | **95% CI** | **Crude OR** | **95% CI** |
| **Child age in months** | <6 | 1 |  | 1 |  |
|  | 6–12 | 1.96 | 0.70–5.50 | 1.19 | .50–2.83 |
|  | 12–23 | 1.72 | 0.65–4.56 | 1.19 | .55–2.57 |
|  | 24–35 | 0.83 | 0.29–2.42 | 0.68 | .32–1.45 |
|  | 36–47 | 1.12 | 0.40–3.11 | 0.90 | .42–1.92 |
|  | 48–59 | 0.92 | 0.29–2.88 | 0.73 | .32–1.67 |
| **Sex of the child** | Male | 1 |  | 1 |  |
|  | Female | 1.02 | 0.56–1.84 | .78 | .56–1.09 |
| **Maternal age** | < 20 | 1 |  | 1 |  |
|  | 20–29 | 1.32 | 0.66–2.65 | .99 | .65–1.51 |
|  | ≥30 | 0.94 | 0.34–2.59 | .93 | .50–1.7 |
| **Religion** | Hindu | 1 |  | 1 |  |
|  | Other | 0.74 | 0.35–1.58 | 1.16 | .62–2.18 |
| **Ethnicity** | Brahmin | 1 |  | 1 |  |
|  | Chhetri | 0.21* | 0.06–0.73 | .33** | .15–.72 |
|  | Madheshi | 1.32 | 0.31–5.66 | 2.02 | .73–5.58 |
|  | Dalit | 0.28 | 0.08–1.04 | .53 | .22–1.25 |
|  | Janajati | 0.54 | 0.15–1.93 | .69 | .30–1.56 |
|  | Newar | 0.57 | 0.04–8.72 | 5.19 | .60–44.95 |
|  | Muslim | 0.83 | 0.13–5.35 | 8.78* | 1.12–69.16 |
| **Maternal education** | No Education | 1 |  |  |  |
|  | Basic | 0.49 | 0.22–1.08 | 1.42 | .87–2.31 |
|  | Secondary | 0.97 | 0.44–2.14 | 1.42 | .85–2.36 |
|  | Higher | 1.09 | 0.22–5.53 | .80 | .26–2.47 |
| **Wealth quintile** | Lowest | 1 |  | 1 |  |
|  | Second | 3.52** | 1.52–8.16 | 2.98*** | 1.80–4.96 |
|  | Middle | 4.06** | 1.72–9.58 | 6.36*** | 3.54–11.43 |
|  | Fourth | 9.10*** | 2.90–28.59 | 12.21*** | 5.96–25.02 |
|  | Highest | 8.62*** | 2.57–28.95 | 15.25*** | 4.89–47.56 |
| **Disadvantages** | Triple | 1 |  | 1 |  |
|  | Double | .71 | .26–1.90 | .83 | .43–1.57 |
|  | Single | .91 | .33–2.51 | 1.76 | .90–3.43 |
|  | No | 1.33 | .39–4.55 | 3.02** | 1.33–6.88 |
| **Province** | Koshi | 1 |  | 1 |  |
|  | Madhesh | 2.94 | 0.88–9.82 | 3.18** | 1.40–7.25 |
|  | Bagmati | 1.56 | 0.45–5.45 | .94 | .43–2.06 |
|  | Gandaki | 0.69 | 0.11–4.33 | 1.13 | .46–2.76 |
|  | Lumbini | 3.58* | 1.07–11.96 | 2.42* | 1.10–5.33 |
|  | Karnali | 0.29* | 0.11–0.78 | .26*** | .13–.52 |
|  | Sudurpaschim | 0.82 | 0.29–2.32 | .54 | .26–1.11 |
| **Place of residence** | Urban | 1 |  | 1 |  |
|  | Rural | 0.68 | 0.36–1.28 | .40*** | .27–.60 |
| **Ecoregion** | Mountain | 1 |  | 1 |  |
|  | Hill | 2.15 | 0.51–9.03 | 3.60*** | 1.99–6.52 |
|  | Terai | 12.34*** | 3.03–50.33 | 21.72*** | 11.15–42.31 |
| **Native language** | Nepali | 1 |  | 1 |  |
|  | Maithili | 4.25*** | 1.85–9.81 | 5.39*** | 2.40–12.14 |
|  | Bhojpuri | 2.27 | .39–13.02 | 2.28* | 1.00–5.16 |
|  | Other | 2.40* | 1.07–5.35 | 1.40 | .83–2.35 |
| **Birth order** | First | 1 |  | 1 |  |
|  | Second | 0.94 | 0.52–1.69 | 1.47 | .98–2.21 |
|  | Third and higher | 1.20 | 0.55–2.63 | .80 | .52–1.23 |

* *p*<.05, ** *p*<.01, *** *p*<.001; CI = confidence interval
