# Supplementary material for: Increasing childhood illnesses (diarrhea and fever) and decreasing care-seeking practices in Nepal: Insights from three most recent Demographic and Health Surveys (2011, 2016 and 2022)
Source: PLOS Glob Public Health. 2025 Dec 11;5(12):e0005651. doi: 10.1371/journal.pgph.0005651 (PMC12698019; doi:10.1371/journal.pgph.0005651)
Supplement: Attachment — Submitted filename: Response to reviewers_final.pdf [file pgph.0005651.s008.pdf]

Point by point response to reviewers

Comments for the editor: This study examines trends and determinants of the prevalence of fever and diarrhea, as well as their care-seeking practices based on socioeconomic and demographic factors in Nepal. The main contribution of the paper is that it focuses on diarrhea and fever among children aged under five years across the three most recent Nepal Demographic Health Surveys (NDHS). I recommend that this paper be accepted after major revision, including additional analysis to be comparable with previous studies or provide justification for not conducting such analyses.

Response: Thank you for the feedback. Relevant studies were further reviewed and included in the manuscript to justify the knowledge gaps and significance of the study, and interpretation of the findings. All feedback from the reviewer and editor have been included in the revision and justification were provided any feedback for any comments.

Comments for both the editor and the author: This paper investigates trends and determinants of the prevalence of fever and diarrhea in Nepal. This study contributed to pulling out a declining trend in care-seeking behavior with an increase in visits to private health facilities. However, the authors should also state the added value of this study and the scientific significance compared to previous literature, given that this study focused mainly on diarrhea and fever. I have several comments as outlined below.

Response: Thank you for the feedback. Relevant studies were further reviewed and included in the manuscript. All feedback from the reviewers and editor have been included in the revision and justification were provided any feedback for any comments. The significance of the study findings was also included and interpreted in the decision section of the revised manuscript.

Abstract: Line 24: Incomplete sentence, and I suggest that the author add a summary of the statistical analysis applied in this study.

Response: Thank you for the comments, they have been corrected in the revised version of the manuscript.

Introduction: The research or knowledge gaps and aims should be more clearly explained by reviewing the existing literature on this topic, particularly studies conducted in Nepal. For example, previous studies have investigated Trends and prevalence of diarrhea in Nepal, such as:

a. Paudel, D., et al. (2020): Determinants of childhood diarrhoea in Nepal: A multilevel analysis of Nepal Demographic and Health Survey 2016. PLoS ONE, 15(11), e0242570.
https://doi.org/10.1371/journal.pone.0242570
b. Ghimire, U., et al. (2018): Trends and inequalities in diarrhoea among children under five years in Nepal: 2001–2016. Tropical Medicine & International Health, 23(10), 1050–1060. https://doi.org/10.1111/tmi.13127

Please elaborate on what has already been done in Nepal in this field and clearly identify the remaining research gaps that this study seeks to address.

Response: Thank you for the feedback and papers. Unfortunately , suggested papers are not identifiable and doi indicate irrelevant papers to this manuscript. We have further reviewed and revised the literature review in the introduction and discussion section. In the introduction section and discussion section, relevant and recent literature was reviewed and incorporated in the manuscript to justify the literature gaps and significance of the study. Relevant studies were included in the revised manuscript[1-5]. This has been detailed in the second paragraph of page 5 of the manuscript, line 103-120 (Clean copy).

Method: This study has not clearly explained how the trend analysis was performed, i.e., which trend test was utilized? I would like to suggest that the author apply a mixed-effect modeling approach in this study. Demographic Health Survey (DHS) data is hierarchical. Individuals are nested within households, which are nested within clusters which are nested within regions. Mixed effects models allow for correlated observations within clusters or groups by including random effects.

Response: We conducted z text for proportion to analyze statistical significance of

trends in the major outcome variables. As in many previous studies, we employed survey logistic regression analysis to determine the associated factors with the outcome variables. Survey logistic regression analysis is DHS data analysis (more than 80% studies conducted using DHS employed the survey logistic regression analysis[6]. To account for the hierarchical structure of the sample design, we used survey logistic regression which applies survey-weighted logistic regression, and it considers the survey's complex design features—specifically clustering, stratification, and sampling weights[7]. Sampling weights provided in the DHS datasets ensure that the estimates are nationally representative. Clustering effect of the sampling design can be adjusted using "svy" command in Stata, taking consideration of sampling weight given in the dataset, which also account the primary sampling units and stratification (provinces)[1]. Stratification was also accounted for by including variables such as province in the model. Furthermore, we assessed multicollinearity using the Variance Inflation Factor (VIF) and evaluated model fit using the Hosmer–Lemeshow test. Detailed elaboration is included in the revised manuscript, page 7, line 166-192.

Results: The inclusion of six figures and four tables makes the manuscript feel quite extensive. The authors may consider streamlining or consolidating some of the figures and tables to enhance clarity and conciseness.

Response: Thank you for the feedback. We have consolidated the figures related to prevalence, care-seeking behavior, and place of care-seeking into a single figure for each symptom (diarrhea and fever). As a result, the revised manuscript now contains two figures and four tables.

Comment Figures: Statistically significant signs should be in all figures' footnotes.

Response: important notations of the figure were included in the footnotes of each figure.

Typo: Line 495: "Fifthly, addressing Addressing"

Response: We have corrected these errors.

Discussion: What is the reason for the increasing percentage of "Not treated" diarrhea in Nepal? Please add an explanation in the discussion section.

Response: Thank you for important feedback. We have reviewed more literature and why there are trends in no treatment of cases for childhood illnesses. The reasons for no treatment of cases have been elaborated in the discussion in the second paragraph of page 22, line 489-511.

References

1. Mohammed MOM: **Prevalence and risk factors associated with under-five years children diarrhea in Malawi: Application of survey logistic regression**. *Heliyon* 2024, **10**(7).
2. Thapa B, Sapkota S, Hu Y: **Predictors of diarrhea among children under 24 months in Nepal: A multilevel analysis of multiple indicator cluster survey 2019**. *PLoS One* 2023, **18**(7):e0287974.
3. Ghimire PR, Agho KE, Renzaho AM, Dibley M, Raynes-Greenow C: **Association between health service use and diarrhoea management approach among caregivers of under-five children in Nepal**. *PloS one* 2018, **13**(3):e0191988.
4. Budhathoki SS, Bhattachan M, Yadav AK, Upadhyaya P, Pokharel PK: **Eco-social and behavioural determinants of diarrhoea in under-five children of Nepal: a framework analysis of the existing literature**. *Tropical Medicine and Health* 2016, **44**(1):7.
5. Malqvist M, Pun A, Raaijmakers H, Kc A: **Persistent inequity in maternal health care utilization in Nepal despite impressive overall gains**. *Global Health Action* 2017, **10**(1):1356083.
6. Dey D, Haque MS, Islam MM, Aishi UI, Shammy SS, Mayen MSA, Noor STA, Uddin MJ: **The proper application of logistic regression model in complex survey data: a systematic review**. *BMC Med Res Methodol* 2025, **25**(1):15.
7. Bhatta DN, Glantz S: **Parental tobacco use and child death: analysis of data from demographic and health surveys from South and South East Asian countries**. *International Journal of Epidemiology* 2019, **48**(1):199-206.
