# Supplementary material for: Increasing childhood illnesses (diarrhea and fever) and decreasing care-seeking practices in Nepal: Insights from three most recent Demographic and Health Surveys (2011, 2016 and 2022)
Source: PLOS Glob Public Health. 2025 Dec 11;5(12):e0005651. doi: 10.1371/journal.pgph.0005651 (PMC12698019; doi:10.1371/journal.pgph.0005651)
Supplement: Attachment — Submitted filename: Reviewer_Report_PGPH-D-25-01861R1.pdf [file pgph.0005651.s009.pdf]

Reviewer Report

Manuscript Title:

Rising childhood illnesses (diarrhea and fever) and shifting care-seeking practices in Nepal: insights from three most recent demographic and health surveys (2011, 2016, and 2022)

Manuscript ID:

PGPH-D-25-01861R1

General Evaluation

This manuscript addresses a timely and policy-relevant topic using nationally representative data from three rounds of the Nepal Demographic and Health Survey (NDHS). It provides valuable insights into the changing burden of common childhood illnesses and evolving care-seeking practices, particularly in the context of growing reliance on private health facilities. The focus on intersectional inequities is commendable and aligns well with global health equity priorities. However, to enhance the scientific rigor, the manuscript would benefit from clearer articulation of its novelty, refinement of the methodological description, and more analytical discussion of results. Substantial revisions are needed before it can be considered for publication.

2. Major Comments

1. Originality and Rationale
- Clarify the paper's novel contribution compared to existing NDHS-based analyses.
- Strengthen the theoretical justification for combining wealth, education, and ethnicity into the marginalization variable.

2. Data and Methods
- Specify the use of survey weights and address comparability across DHS rounds.
- Provide detailed description of marginalization variable construction and validation.
- Focus the regression section on application rather than general definitions.

3. Results
- Condense repetitive sections and move detailed outputs to supplementary materials.
- Improve figures by adding confidence intervals and consistent legends.

4. Discussion
- Focus more on interpretation than restating results.
- Expand discussion on causes of increased private facility use and policy implications.

5. Policy Implications

- Provide specific actionable strategies based on your findings.

6. Limitations

- Mention recall bias, and survey consistency issues.

3. Minor Comments

- Include numerical trends in the abstract.
- Conduct light language editing.
- Move long tables to supplementary materials.
- Ensure consistency in terminology (e.g., public vs private HFs).
- Update references (2022–2024 studies).
- Maintain consistent statistical notation for AORs and p-values.

4. Summary Recommendation

Decision: Major Revision
