# Supplementary material for: Increasing childhood illnesses (diarrhea and fever) and decreasing care-seeking practices in Nepal: Insights from three most recent Demographic and Health Surveys (2011, 2016 and 2022)
Source: PLOS Glob Public Health. 2025 Dec 11;5(12):e0005651. doi: 10.1371/journal.pgph.0005651 (PMC12698019; doi:10.1371/journal.pgph.0005651)
Supplement: Attachment — Submitted filename: Peer-review report.docx [file pgph.0005651.s007.docx]

**9^th^ August 2025**

**Peer-review summary for the manuscript titled**

**“Trends and determinants of prevalence and care-seeking of diarrhea and fever among children under five in Nepal: an analysis of the most recent Nepal Demographic and**

**Health Surveys”**

**Comments for the editor**

This study examines trends and determinants of the prevalence of fever and diarrhea, as well as their care-seeking practices based on socioeconomic and demographic factors in Nepal. The main contribution of the paper is that it focuses on diarrhea and fever among children aged under five years across the three most recent Nepal Demographic Health Surveys (NDHS). I recommend that this paper be accepted after major revision, including additional analysis to be comparable with previous studies or provide justification for not conducting such analyses.

**Comments for both the editor and the author**

This paper investigates trends and determinants of the prevalence of fever and diarrhea in Nepal. This study contributed to pulling out a declining trend in care-seeking behavior with an increase in visits to private health facilities. However, the authors should also state the added value of this study and the scientific significance compared to previous literature, given that this study focused mainly on diarrhea and fever. I have several comments as outlined below.

**Minor comments:**

**Abstract**

1. Line 24: Incomplete sentence
2. I suggest that the author add a summary of the statistical analysis applied in this study.

**Results**

The inclusion of six figures and four tables makes the manuscript feel quite extensive. The authors may consider streamlining or consolidating some of the figures and tables to enhance clarity and conciseness.

**Figures**

3. Statistically significant signs should be in all figures' footnotes.

**Typo**

4. Line 495: “Fifthly, addressing Addressing”

**Discussion**

5. What is the reason for the increasing percentage of "Not treated" diarrhea in Nepal? Please add an explanation in the discussion section.

**Major Comments:**

**Introduction**

6. The research or knowledge gaps and aims should be more clearly explained by reviewing the existing literature on this topic, particularly studies conducted in Nepal. For example, previous studies have investigated Trends and prevalence of diarrhea in Nepal, such as:
a. Paudel, D., et al. (2020): Determinants of childhood diarrhoea in Nepal: A multilevel analysis of Nepal Demographic and Health Survey 2016. PLoS ONE, 15(11), e0242570.

https://doi.org/10.1371/journal.pone.0242570

b. Ghimire, U., et al. (2018): Trends and inequalities in diarrhoea among children under five years in Nepal: 2001–2016. Tropical Medicine & International Health, 23(10), 1050–1060. https://doi.org/10.1111/tmi.13127

Please elaborate on what has already been done in Nepal in this field and clearly identify the remaining research gaps that this study seeks to address.

**Method**

7. This study has not clearly explained how the trend analysis was performed, i.e., which trend test was utilized?
8. I would like to suggest that the author apply a mixed-effect modeling approach in this study. Demographic Health Survey (DHS) data is hierarchical. Individuals are nested within households, which are nested within clusters, which are nested within regions. Mixed effects models allow for correlated observations within clusters or groups by including random effects.
