# Supplementary material for: Increasing childhood illnesses (diarrhea and fever) and decreasing care-seeking practices in Nepal: Insights from three most recent Demographic and Health Surveys (2011, 2016 and 2022)
Source: PLOS Glob Public Health. 2025 Dec 11;5(12):e0005651. doi: 10.1371/journal.pgph.0005651 (PMC12698019; doi:10.1371/journal.pgph.0005651)
Supplement: Attachment — Submitted filename: Response to reviewers_R2.docx [file pgph.0005651.s010.docx]

**Point by point response to the reviewers**

Rising childhood illnesses (diarrhea and fever) and shifting care-seeking practices in Nepal: insights from three most recent demographic and health surveys (2011, 2016, and 2022)

This manuscript addresses a timely and policy-relevant topic using nationally representative data from three rounds of the Nepal Demographic and Health Survey (NDHS). It provides valuable insights into the changing burden of common childhood illnesses and evolving care-seeking practices, particularly in the context of growing reliance on private health facilities. The focus on intersectional inequities is commendable and aligns well with global health equity priorities. However, to enhance the scientific rigor, the manuscript would benefit from clearer articulation of its novelty, refinement of the methodological description, and more analytical discussion of results. Substantial revisions are needed before it can be considered for publication.

Response : thank you for this general feedback, we appreciate for your time and effort for reading and providing feedback on our manuscript.

**Comment:** Originality and Rationale - Clarify the paper’s novel contribution compared to existing NDHS-based analyses. - Strengthen the theoretical justification for combining wealth, education, and ethnicity into the marginalization variable.

**Response:** The introduction section was revised to include the study's rationale and significance. Furthermore, novel insights from this paper were added to justify the study. The theoretical justification, particularly concerning structural inequity and the role of structural factors like wealth status, education, and ethnicity, was also incorporated into the introduction. The methods section explains how these three variables were used to determine marginalisation status, referencing previous literature in the field, including recent work by the first author.

**Comment:** Data and Methods - Specify the use of survey weights and address comparability across DHS rounds. Provide detailed description of marginalization variable construction and validation. Focus the regression section on application rather than general definitions.

**Response:** We adjusted for the clustering effects of the complex sampling survey design using the SVY command for strata (which considers the strata, primary sampling unit, and sampling weight assigned to each individual sample in the dataset) for each individual survey year. This was not a pooled analysis so we conducted the proportion analysis for each outcome variable for each survey, and a significance test was performed using the prtesti command in Stata. The determinants of the associated factors were examined using the NDHS 2022. The survey weights were calculated using the sampling weight provided in the dataset (considering the strata and primary sampling units). Details of the code for this analysis are publicly available in the GitHub repository: [DHS-Analysis-Code/Analysis_Reports/FA158_childhealthequity/FA158_Child Health_19.07.24.do at main · DHSProgram/DHS-Analysis-Code · GitHub](https://github.com/DHSProgram/DHS-Analysis-Code/blob/main/Analysis_Reports/FA158_childhealthequity/FA158_Child%20Health_19.07.24.do)

Response: A detailed description of the marginalization variable was provided in the methods section (subsection: Independent Variable). We referenced previous studies on how it was created, including four publications from the first authors of this manuscript [1-6].

The methods section has been revised to include details on how the regression analysis was performed..

**Comment:** Results - Condense repetitive sections and move detailed outputs to supplementary materials. - Improve figures by adding confidence intervals and consistent legends.

**Response:** Supplementary materials were included to support the results section, specifically the descriptive analysis and the output of the bivariable analysis. The figures were edited for clarity and focus.

**Comment:** Discussion - Focus more on interpretation than restating results. - Expand discussion on causes of increased private facility use and policy implications.

**Response:** The discussion section has included the interpretation of the findings and the recommendations, particularly regarding the increased care-seeking in the private HFs for childhood illnesses. The policy implications were also revised, with a focus on the potential for increased financial hardship when paying for private services in the context of a lack of a health insurance program.

**Comment:** Policy Implications - Provide specific actionable strategies based on your findings. Limitations - Mention recall bias, and survey consistency issues

**Response :** Action-oriented strategies were incorporated into the policy implications subsection. The limitations were revised and explained, including the potential for recall bias in the survey and the general data collection process of the NDHS.

**Comments:**

- Include numerical trends in the abstract.
- Conduct light language editing.
- Move long tables to supplementary materials.
- Ensure consistency in terminology (e.g., public vs private HFs).
- Update references (2022–2024 studies).
- Maintain consistent statistical notation for AORs and p-values.

**Responses:**

- Numerical values for the trends were added to the abstract.
- Comprehensive language editing was performed on the manuscript and verified in the final cleaned version.
- Necessary data have been included as supplementary materials, with only the most significant findings presented in the main manuscript.
- The manuscript was reviewed and edited to ensure consistency in terminology throughout.
- Appropriate and relevant references were updated, including papers published after 2022.
- Consistency in statistical notation was ensured across the manuscript.

**References**

1.	Khatri R, Dulal KP, Timelsena K, Tamrakar M, Rosenberg R, Tuladhar S: **Equity Analysis of Maternal Health Services in Nepal: Trends and Determinants, 2011–2022 Nepal DHS Surveys**. In*.*; 2024.

2.	Khatri R, Dulal KP, Timelsena K, Tamrakar M, Tuladhar S, Rosenberg R: **Prevalence of and Care Seeking for Diarrhea and Fever Among Children Under 5: An Intersectional Equity Analysis, 2011–2022 Nepal DHS Surveys**. In*.* Kathmandu, Nepal; 2024.

3.	Khatri RB: **Towards equity of maternal and newborn health services in Nepal. PhD thesis.** . *PhD Thesis.* The University of Queensland, Australia; 2021.

4.	Khatri RB, Alemu Y, Protani MM, Karkee R, Durham J: **Intersectional (in) equities in contact coverage of maternal and newborn health services in Nepal: insights from a nationwide cross-sectional household survey**. *BMC Public Health* 2021, **21**(1):1098.

5.	Endalamaw A, Gilks CF, Khatri RB, Assefa Y: **Intersectional inequity in knowledge, attitude, and testing related to HIV in Ethiopia: People with multiple disadvantages are left behind**. *PLOS Global Public Health* 2024, **4**(8):e0003628.

6.	Devasenapathy N, Neogi SB, Soundararajan S, Ahmad D, Hazra A, Ahmad J, Mann N, Mavalankar D: **Association of antenatal care and place of delivery with newborn care practices: evidence from a cross-sectional survey in rural Uttar Pradesh, India**. *J Health Popul Nutr* 2017, **36**(1):30.
